# Validation of the Official Slovak Version of the Unified Dyskinesia Rating Scale (UDysRS)

**DOI:** 10.1155/2015/674796

**Published:** 2015-07-02

**Authors:** Matej Skorvanek, Michal Minar, Milan Grofik, Katarina Kracunova, Vladimir Han, Frantisek Cibulcik, Jan Necpal, Ladislav Gurcik, Peter Valkovic

**Affiliations:** ^1^Department of Neurology, Safarik University, 04001 Kosice, Slovakia; ^2^Department of Neurology, University Hospital of L. Pasteur, 04001 Kosice, Slovakia; ^3^2nd Department of Neurology, Faculty of Medicine, Comenius University, 83305 Bratislava, Slovakia; ^4^Department of Neurology, Comenius University, 03659 Martin, Slovakia; ^5^Department of Neurology, Slovak Medical University, 83303 Bratislava, Slovakia; ^6^Department of Neurology, Zvolen Hospital, 96001 Zvolen, Slovakia; ^7^Department of Neurology, VNsP Levoca, 05401 Levoca, Slovakia; ^8^Institute of Normal and Pathological Physiology, Slovak Academy of Sciences, 81371 Bratislava, Slovakia

## Abstract

After successful clinimetric testing of the Unified Dyskinesia Rating Scale (UDysRS), a program for translation and validation of non-English versions of the UDysRS was initiated. The aim of this study was to validate and confirm the factor structure of the Slovak translation of the UDysRS. We examined 251 patients with Parkinson's disease and dyskinesia using the Slovak version of the UDysRS. The average age of our sample was 65.2 ± 9.2 years and average disease duration was 10.9 ± 5.0 years. Slovak data were compared using confirmatory factor analysis with the Spanish data. To be designated as the official Slovak UDysRS translation, the comparative fit index (CFI) had to be ≥0.90 relative to the Spanish language version. Exploratory factor analysis was performed to explore the underlying factor structure without the constraint of a prespecified factor structure. For all four parts of the Slovak UDysRS, the CFI, in comparison with the Spanish language factor structure, was ≥0.98. Isolated differences in the factor structure of the Slovak UDysRS were identified by exploratory factor analysis compared with the Spanish version. The Slovak version of the UDysRS was designated as an official non-English translation and can be downloaded from the website of the International Parkinson and Movement Disorder Society.

## 1. Introductions

Drug-induced dyskinesia is one of the most troublesome therapeutic issues in Parkinson's disease (PD) with a significant impact on patients' quality of life [1–3]. Several existing dyskinesia rating scales in PD have focused on one or more of their attributes, including their anatomical distribution, duration, intensity, phenomenology, disability, and patient perceptions. However, as none of these scales covered all of these issues, the Unified Dyskinesia Rating Scale (UDysRS) has been developed to provide a comprehensive rating tool of dyskinesia in Parkinson's disease [4]. The UDysRS is composed of four parts: (1) Historical Disability (patient perceptions) of ON-Dyskinesia impact (11 items, maximum 44 points); (2) Historical Disability (patient perception) of OFF-Dystonia impact (4 items, maximum 16 points); (3) objective impairment (dyskinesia severity, anatomical distribution over seven body regions, and type (choreic and/or dystonic) based on four activities observed or video recorded (7 items, maximum 28 points)); (4) objective disability based on Part 3 activities (4 items, maximum 16 points). The UDysRS has shown acceptable internal consistency, inter- and intrarater reliability, and temporal stability [4, 5]. The UDysRS has also shown the highest effect size for detecting treatment-related change compared to other existing clinical scales evaluating dyskinesia in PD [6]. To enhance a uniform administration of the UDysRS a DVD-based training program along with a certification exercise available online has been developed [7]. Since dyskinesia therapies involve multicenter studies, having a scale that is validated in multiple non-English languages is pivotal to international efforts targeting dyskinesia. In this regard, a uniform clinimetric testing program for non-English translations of the UDysRS has been developed [8]. The aim of this study was the clinimetric testing and validation of the Slovak version of the UDysRS.

## 2. Methods

### 2.1. Design

This was a Slovak national, multicenter, cross-sectional study.

### 2.2. Raters and Patients

Neurologists specialized in movement disorders were contacted in person or by email by the national coordinator (MS). All 8 contacted Neurology Departments agreed to participate in the study; patients were examined by 11 raters. The study enrolled consecutive patients with Parkinson's disease and dyskinesia diagnosed according to the UK PD Brain Bank Criteria [9] regardless of their age, disease stage, and cognitive status. Sampling was monitored in order to ensure that all levels of dyskinesia severity were adequately represented. The study was approved by the ethics committees of Safarik University in Kosice and the Comenius University in Bratislava. All patients participated voluntarily and gave written informed consent prior to the study. The investigation was performed according to the Declaration of Helsinki. Deidentified data, without patient names or medical record numbers, were transferred to the analytic team via a secure website.

### 2.3. Procedure

The UDysRS was translated into Slovak and then back-translated into English using two independent teams. Cognitive pretesting was conducted on the Slovak version and required modifications to the structure or wording of the translation were completed. The final Slovak version was administered to a large cohort of Parkinson's disease patients whose native language is Slovak. To conduct the cognitive pretesting and data entry of the large cohort, a cognitive pretesting packet and a data entry guide were translated into Slovak. The data from the large Slovak cohort were analyzed to assess the confirmatory factor structure to the Spanish UDysRS (the Reference Standard) [10]. Secondary analyses included an exploratory factor analysis, independent of the Reference Standard.

### 2.4. Cognitive Pretesting

Cognitive pretesting is a qualitative approach to assessing task difficulty for examiner and respondent, respondent interest, attention span, discomfort, and comprehension [11]. Cognitive pretesting was conducted for the instructions to raters and patients as well as for items and response options for Time Spent with On-Dyskinesia, Eating Tasks, Exciting or Emotional Setting, Time Spent with Off-Dystonia, Effects of Off-Dystonia Separate from Pain, Effects of Pain from Off-Dystonia, and Objective Evaluation of Dyskinesia Disability. Based on the results of the initial cognitive pretesting, other round(s) of translation and back translation and cognitive pretesting could be required. Once cognitive pretesting was taken into account, the final translation was obtained.

### 2.5. Statistical Analyses

Mplus, Version 7, was used to do the confirmatory and exploratory factor analyses because this program is designed specifically for analyzing categorical variables [12]. Unweighted least squares (ULS) approach to factor estimation that minimizes the sum of squared differences between observed and estimated correlation matrices not counting diagonal elements was used. To assist in interpretation of the factors an orthogonal VARIMAX rotation that constrains the factors to be uncorrelated was used. As done in the other language translation validation studies, Question  1 (Time of ON-Dyskinesia) and Question  12 (Time of OFF-Dystonia) were omitted from the factor analyses and were considered as descriptive indices rather than measures of impairment or disability.

As the primary analysis of the Slovak data, a confirmatory factor analysis (CFA), comparing the Slovak data to those from the Reference Standard UDysRS data, was conducted. We determined if the factor structure for the Reference Standard UDysRS could be confirmed in the data collected using the Slovak translation. This was the primary question of interest. The CFA results based on the Comparative Fit Index (CFI) were evaluated. To confirm a good fit between the Slovak and Spanish UDysRS, the CFI was required to be 0.90 or greater. Mean and variance adjusted weighted least square (WLSMV) estimator was used to confirm model fit. We also used the root mean square error of approximation (RMSEA) to check the goodness of fit. The RMSEA is a population-based index that relies on the noncentral *χ* ^2^ distribution, which is the distribution of the fitting function when the fit of the model is not perfect, with values less than or equal to 0.10 indicating an acceptable index.

As a secondary analysis an exploratory factor analysis (EFA) to explore the underlying factor structure for the Slovak translation, without constrain of a prespecified factor structure, using an unweighted least squares (ULS) approach was conducted. We used a SCREE plot from the Slovak data in reference to the Reference Standard SCREE plot to choose the number of factors retained for UDysRS. The subjective SCREE test [13] uses a scatter plot of eigenvalues plotted against their ranks with respect to magnitude, to extract as many factors as there are eigenvalues that fall before the last large drop (i.e., an “elbow” shape) in the plot. Once the factors were chosen, an item was retained in a factor if the factor loading for the item was 0.40 or greater. To assist interpretation of the factors, an orthogonal CF-VARIMAX rotation was used which sets the factors to be uncorrelated.

The sample size requirement for the translation study was based on the need for 7 to 10 subjects per item of the questionnaire in order to perform the tasks needed to validate the instrument. Because there are 26 items on the UDysRS, a sample of at least 250 was required.

## 3. Results

### 3.1. Baseline Characteristics

**Figure 1:**
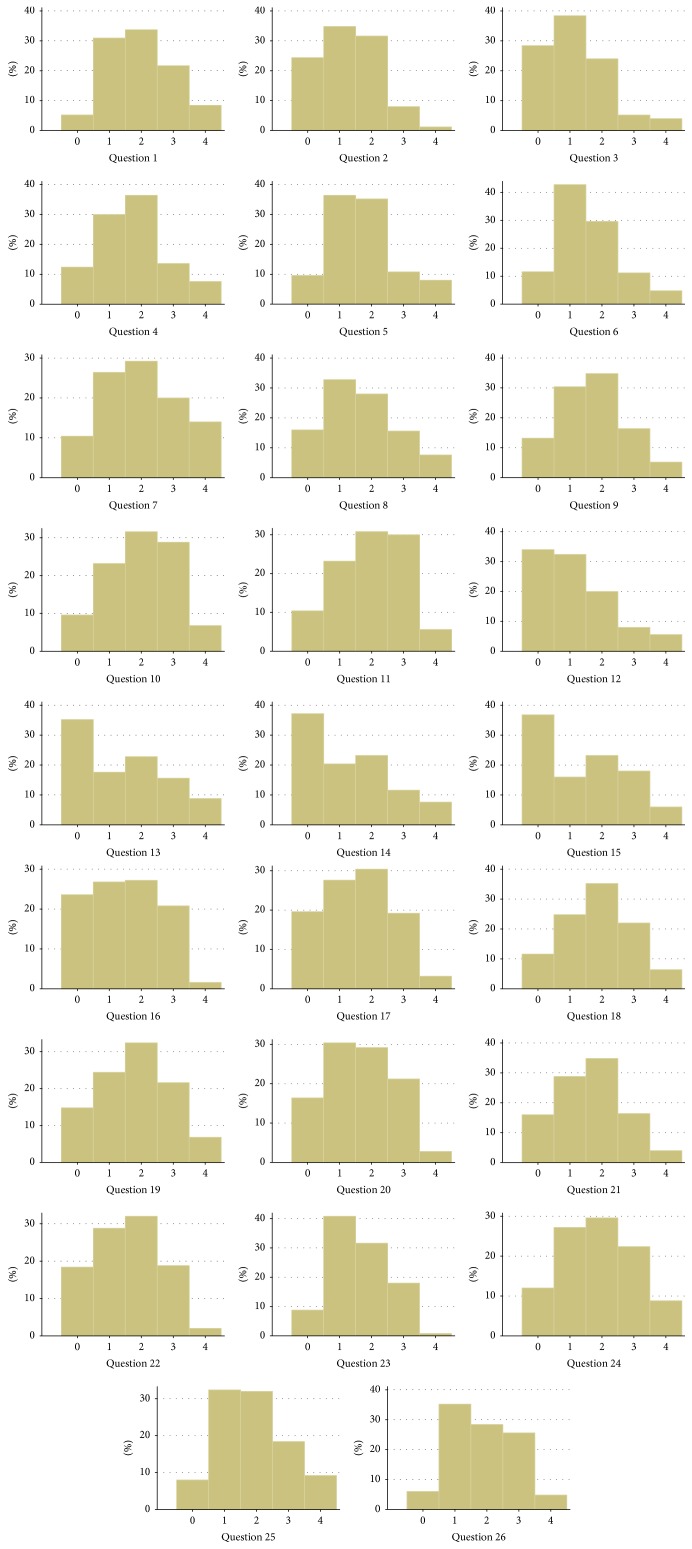
Histograms for each question in the Slovak dataset.   ^*∗*^Questions  1  and  12 are excluded from the factor analysis.

The demographic characteristics of the Slovak patients are shown in Table 1. The Slovak dataset included 251 native Slovak-speaking patients with dyskinesia who were examined using the UDysRS. Except for one Asian all the patients recruited are non-Hispanic white.  Figure 1 gives the distributions of answers to each question.

### 3.2. Cognitive Pretesting

Cognitive pretesting was completed with 4 raters and 10 patients. There were no identified problems with the ease of use, comfort for individual items, or comprehension of items. Three structural problems were noted (misspelling of one word; incorrect spacing on two items) and corrected without further cognitive pretesting.

### 3.3. Confirmatory Factor Analysis (CFA)

Mplus performs listwise deletion of cases with any missing data. That is, any case with one or more missing data points is omitted entirely from analyses. Thus the sample size in factor analysis is 250.  Table 2 displays the CFA for both the Slovak dataset and Reference Standard. The CFI for the Slovak translation, in comparison with the Reference Standard factor structure, was 0.98. Our prespecified criterion was a CFI of 0.90 or greater. Hence, we conclude that the prespecified factor structure was confirmed in the Slovak dataset.

### 3.4. Exploratory Factor Analysis (EFA)

**Figure 2:**
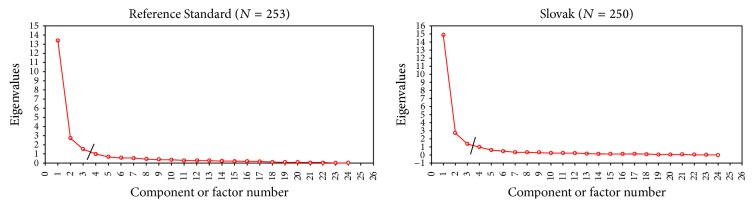
SCREE plot for the Reference Standard and Slovak datasets without “Time Spent with On-Dyskinesia” and “Time Spent with Off-Dystonia.  ^*∗*^Questions on “Time Spent with On-Dyskinesia” and “Time Spent with Off-Dystonia” were not considered in the SCREE plots as they were not included in the factor analysis.

Our EFA analysis for the Slovak dataset differs from the EFA of the Reference Standard dataset in some areas.  Table 3 shows the results of the exploratory factor analysis for all patients of the Reference Standard and Slovak UDysRS without “Time Spent with On-Dyskinesia” and “Time Spent with Off-Dystonia.” The SCREE plots are given in Figure 2.

## 4. Discussion

Levodopa-induced dyskinesia is one of the major determinants of worse quality of life in Parkinson's disease [14]. In order to establish the UDysRS as the international gold standard tool for the assessment of dyskinesia in PD, it is crucial that properly tested non-English translations are made available. The overall factor structure of the Slovak version was consistent with that of the Reference Standard based on the CFIs for all models of the UDysRS in the confirmatory factor analysis (CFI = 0.98). The Slovak scale was confirmed to share a common factor structure with the Reference Standard. Therefore, the Slovak version of the UDysRS presented here fulfills the criteria to be designated as an official translation of the UDysRS and as such is now available for use in further studies. In the exploratory factor analysis, where variability from sample to sample is expected, we identified isolated item differences in factor structure between the Slovak and Reference Standard versions of the UDysRS. These subtle differences may relate to differences in sample composition or variations in disease status.

Validation of the Slovak version of the UDysRS is part of an IPMDS organized clinimetric testing program for non-English translations of the Movement Disorder Society-Unified Parkinson's Disease Rating Scale (MDS-UPDRS) and the UDysRS [8]. In order to enhance a uniform application of the UDysRS, a DVD-based and online training program with instructions, patient examples, and certification exercise has been developed [7]. Besides the Slovak version of the UDysRS, the global program now includes also the Chinese, French, German, Hungarian, and Spanish translations which are available online (http://www.movementdisorders.org/publications/rating_scales/) and other translations are in development. Other language teams are encouraged to participate in this effort (contact: CG Goetz, cgoetz@rush.edu). As future large-scale clinical trials using the UDysRS will necessarily recruit centers from countries where official translations are available, this effort has practical implications for future research programs. The UDysRS and its official translations are owned by the IPMDS and for future clinical trials the access rights can be arranged through the IPMDS.

The scale provides patients' subjective as well as raters' objective rating of dyskinesia making it a comprehensive tool for their assessment. However, due to this complex evaluation the length of scale administration is longer than in the other existing scales. Drug-induced dyskinesia in PD is seen especially during action and therefore provocation maneuvers such as speaking, drinking, walking, and dressing are used during objective dyskinesia evaluations. Nevertheless, the scale can be easily administered in standard clinical settings.

Despite small Slovak population compared to other language groups enrolled in the UDysRS program, the translation and validation process of the Slovak UDysRS was finished within 12 months from its initiation. This was achieved by successful enrollment of raters from most Slovak centers specialized in movement disorders with good cooperation also in previous joint projects. Raters were encouraged to enroll consecutive patients with dyskinesia. Also, the study coordinator actively monitored the enrollment process and regularly contacted and informed individual raters about its progress. In order to optimize the process and speed up successful accomplishment of future similar translation programs, respective validation teams should maximize their multicenter approach and organization of the team including regular communication with individual raters.

## 5. Conclusions

The overall factor structure of the Slovak version was consistent with that of the Reference Standard based on the CFIs for all models of the UDysRS in the confirmatory factor analysis (CFI = 0.98). The Slovak scale was confirmed to share a common factor structure with the Reference Standard. Therefore, this version can be designated as the official Slovak version of the UDysRS. In the exploratory factor analysis, where variability from sample to sample is expected, we identified isolated item differences in factor structure between the Slovak and Reference Standard versions of the UDysRS. These subtle differences may relate to differences in sample composition or variations in disease status.

## Figures and Tables

**Table 1:** Demographic characteristics of the study sample.

Patients	Total	Male		Age		Years of dyskinesia		Years of Parkinson	
		*N*	%	Mean	Std.	Mean	Std.	Mean	Std.
Slovak	251^*∗*^	126	50.2	65.2	9.2	4.0	3.0	10.9	5.0
Reference Standard	253	122	48.2	69.2	10.5	4.9	4.6	12.5	6.8

^*∗*^One patient with incomplete data was excluded from the factor analysis.

**Table 2:** CFA model fit^*∗*^.

Slovak	CFI = 0.98, RMSEA = 0.10 (250 patients)

Reference Standard	CFI = 0.98, RMSEA = 0.08 (247 patients)

^*∗*^CFI, comparative fit index; RMSEA, root mean square error of approximation.

**Table 3:** Exploratory factor structures of the UDysRS without “Time Spent with On-Dyskinesia” and “Time Spent with Off-Dystonia” for all Slovak patients (*N* = 250).

	Item	Item factor loading	
		Reference Standard	Slovak
Factor 1	*Percent variance *	*56.0 *	*56.0 *
	Speech	0.63	0.69
	Chewing/swallowing	0.65	0.74
	Eating tasks	0.77	0.79
	Dressing	0.85	0.82
	Hygiene	0.81	0.81
	Hand writing	0.78	0.76
	Doing hobbies/activities	0.74	0.78
	Walking/balance	0.75	0.68
	Public/social	0.7	0.75
	Exciting situations	0.71	0.71

Factor 2	*Percent variance *	*11.5 *	*11.5 *
	Face	0.73	0.69
	Neck	0.76	0.70
	Right hand/arm/shoulder	0.69	0.83
	Left hand/arm/shoulder	0.66	0.74
	Trunk	0.75	0.76
	Right foot/leg/hip	0.66	0.76
	Left foot/leg/hip	0.67	0.73
	Communication	0.79	0.70
	Drinking	0.78	0.70
	Dressing	0.7	0.71
	Ambulation (walking)	0.65	0.77

Factor 3	*Percent variance *	*6.5 *	*6.5 *
	Dystonia effects on activities (not pain)	0.89	0.94
	Effect of Pain from dystonia	0.98	0.98
	Dystonia pain severity	0.93	0.93

## Appendix

*Slovak UDysRS Validation Team*: Matej Skorvanek, Kosice; Vladimir Han, Kosice; Michal Minar, Bratislava; Peter Valkovic, Bratislava; Katarina Kracunova, Bratislava; Frantisek Cibulcik, Bratislava; Milan Grofik; Martin; Branislav Vesely, Nitra; Jan Necpal, Zvolen; Ladislav Gurcik, Levoca; Lucia Surcova, Spisska Nova Ves.
